# Coronavirus Infection-Associated Cell Death Signaling and Potential Therapeutic Targets

**DOI:** 10.3390/molecules26247459

**Published:** 2021-12-09

**Authors:** Rittibet Yapasert, Patompong Khaw-on, Ratana Banjerdpongchai

**Affiliations:** 1 Department of Biochemistry, Faculty of Medicine, Chiang Mai University, Chiang Mai 50200, Thailand; rittibet_ya@cmu.ac.th; 2 Faculty of Nursing, Chiang Mai University, Chiang Mai 50200, Thailand; patompong.kh@cmu.ac.th

**Keywords:** coronavirus, SARS-CoV-2, COVID-19, cell death, targeted therapy, natural compounds

## Abstract

COVID-19 is the name of the disease caused by the severe acute respiratory syndrome coronavirus 2 (SARS-CoV-2) infection that occurred in 2019. The virus–host-specific interactions, molecular targets on host cell deaths, and the involved signaling are crucial issues, which become potential targets for treatment. Spike protein, angiotensin-converting enzyme 2 (ACE2), cathepsin L-cysteine peptidase, transmembrane protease serine 2 (TMPRSS2), nonstructural protein 1 (Nsp1), open reading frame 7a (ORF7a), viral main protease (3C-like protease (3CLpro) or Mpro), RNA dependent RNA polymerase (RdRp) (Nsp12), non-structural protein 13 (Nsp13) helicase, and papain-like proteinase (PLpro) are molecules associated with SARS-CoV infection and propagation. SARS-CoV-2 can induce host cell death via five kinds of regulated cell death, i.e., apoptosis, necroptosis, pyroptosis, autophagy, and PANoptosis. The mechanisms of these cell deaths are well established and can be disrupted by synthetic small molecules or natural products. There are a variety of compounds proven to play roles in the cell death inhibition, such as pan-caspase inhibitor (z-VAD-fmk) for apoptosis, necrostatin-1 for necroptosis, MCC950, a potent and specific inhibitor of the NLRP3 inflammasome in pyroptosis, and chloroquine/hydroxychloroquine, which can mitigate the corresponding cell death pathways. However, NF-κB signaling is another critical anti-apoptotic or survival route mediated by SARS-CoV-2. Such signaling promotes viral survival, proliferation, and inflammation by inducing the expression of apoptosis inhibitors such as Bcl-2 and XIAP, as well as cytokines, e.g., TNF. As a result, tiny natural compounds functioning as proteasome inhibitors such as celastrol and curcumin can be used to modify NF-κB signaling, providing a responsible method for treating SARS-CoV-2-infected patients. The natural constituents that aid in inhibiting viral infection, progression, and amplification of coronaviruses are also emphasized, which are in the groups of alkaloids, flavonoids, terpenoids, diarylheptanoids, and anthraquinones. Natural constituents derived from medicinal herbs have anti-inflammatory and antiviral properties, as well as inhibitory effects, on the viral life cycle, including viral entry, replication, assembly, and release of COVID-19 virions. The phytochemicals contain a high potential for COVID-19 treatment. As a result, SARS-CoV-2-infected cell death processes and signaling might be of high efficacy for therapeutic targeting effects and yielding encouraging outcomes.

## 1. Introduction

Since December 2019, a severe acute respiratory syndrome coronavirus 2 (SARS-CoV-2) epidemic has emerged, posing a global public health threat never seen before [1,2]. This virus caused over 239 million infection cases and 4.88 million deaths till 14 October 2021 [3]. Acute respiratory distress syndrome and severe cytokine release syndrome are the main causes of COVID-19 mortality [4,5]. It shares similarities with previous severe acute respiratory syndrome CoV infections such as severe acute respiratory syndrome (SARS) and Middle East respiratory syndrome (MERS) [6]. COVID-19 is an acronym that stands for coronavirus disease of 2019. SARS-CoV-2 was classified in Coronaviruses (CoVs), which are a group of enveloped, single-stranded positive-sense RNA viruses with club-like spikes on their surface [7,8]. During replication, CoVs are prone to mutation and recombination, which has led to the diversity and uncontrollability of coronaviruses by the former developed vaccines. SARS-CoV-2 infects the host cell via interacting of its spike protein with angiotensin-converting enzyme 2 (ACE2) receptors on a host cell membrane [9,10]. Virus entry into cells can directly induce host cell death after the virions are enormously produced. Additionally, an excessive release of cytokines and chemokines, known as a COVID-19-related cytokine storm, is also a cause of host cell death [11].

Fever, dry cough, and lethargy are the most common respiratory symptoms of a patient infected with coronavirus. Other symptoms include loss of taste and smell, nasal congestion, conjunctivitis, sore throat, muscular and joint discomfort, diarrhea, shortness of breath, and hyperthermia in certain individuals. Most infected people have mild to moderate symptoms [12]; however, approximately 33 percent of hospitalized COVID-19 patients develop acute respiratory distress syndrome (ARDS) [13]. Currently, there is no standard treatment available. Pre-existing medications could be repositioned as a quick and appealing strategy with well-known safety, features, and dosage. Several medications have been tested for efficacy and safety in the treatment of COVID-19, with most of them still in clinical trials [14].

Aside from vaccine research, much work has been performed towards finding effective COVID-19 prophylactics for high-risk populations, but only a few studies have yielded positive results. Several clinical examples and in vivo studies have lately suggested that some anti-inflammation and anti-virus medications could be used as preventive possibilities [15]. Natural products and herbal medications have been utilized to prevent and treat viral infection for thousands of years in oriental traditional medicine [16]. Those compounds have a high efficiency and a low toxicity. Herbal medicine is undeniably a valuable resource for therapeutic development, and its low toxicity makes it a viable prophylactic candidate against COVID-19 [17,18]. The antiviral action mechanisms of these natural agents on the influence of the viral life cycle, such as viral entrance, replication, assembly, and release, as well as virus–host-specific interactions, have been studied intensively over the last few decades [19]. In this review, we aim to provide an update on the molecular mechanisms of coronavirus 2 infection process and SARS-CoV2-induced cell death and signaling, new therapeutic strategies, and remarkable natural compounds regarding prevention and treatment of COVID-19.

## 2. Life Cycle of SARS-CoV-2 and the Viral Proteins Involved in the Infection

The primary site of attachment and replication of inhaled SARS-CoV-2 is epithelial cells in the nasal cavity. Then virus spreads and migrates across the respiratory tract, following the conducting airways [20]. Virus can infect the host cell by interacting of its spike protein with angiotensin-converting enzyme receptor 2 (ACE2) on a host cell membrane [21]. It requires the host cell’s transmembrane protease, serine 2 (TMPRSS2), to activate the spike protein and cleave the ACE2 receptor, which then allows it to attach to the host cell membrane and induce viral and plasma membrane fusion. In comparison with ACE2 expression in lung-specific pulmonary alveolar type II cells, ACE2 is abundantly expressed in the bladder, ileum, kidney, and liver. However, the renin–angiotensin system and the PPAR signaling pathway, as well as a few basic metabolic and influential pathways, such as insulin resistance, play an important role in enhancing the infection following its entry via ACE2 [22]. Viral RNA is released into the host cell after entry, and viral polyproteins are translated using the ribosome of the host cell. Polyproteins (PP) PP1a and PP1ab are formed during translation and are then cleaved by viral proteases, papain-like protease (PLPRO), and 3C-like protease (3CLPRO) to produce functional non-structural proteins (Nsp). Two types of proteins are encoded by SARS-CoV-2 genomic RNA. Nsp and structural proteins are essential for viral RNA synthesis and virion assembly, respectively [23]. The RNA-dependent RNA polymerase (RdRp) (NSP12), helicase (NSP13), and other subunits such as NSP7 and NSP8 comprise the replicase–transcriptase complex (RTC). The complex also transcribes a viral genome template containing negative-sense genes, as well as progeny genome and subgenomic RNA as intermediary products, before directing RdRp to transcribe positive-sense mRNAs [24]. After transcription and translation, subgenomic proteins form structural and accessory proteins such as M, S, and E proteins, which are then encased in the endoplasmic reticulum and transported to the endoplasmic reticulum–Golgi intermediate compartment (ERGIC). The viral genome can then bind the N protein directly to create the nucleocapsid, which is then insulated into the ERGIC. Finally, nucleocapsids and numerous other structural proteins combine to create vesiculated virion particles, which are then exocytosed out of the cell in the form of virions [25,26].

SARS-CoV and SARS-CoV-2 are both zoonotic coronaviruses belonging to the Betacoronavirus genus [27,28]. The novel SARS-CoV-2 genome was found to share 82 percent nucleotide similarity with SARS-CoV. The membrane, envelope, spike, nucleoprotein, and orf1a/b polyproteins clustered closely together, according to phylogenetic research [29]. It was also confirmed that, similar to SARS-CoV, the angiotensin-converting enzyme 2 (ACE2) is the major host receptor for SARS-CoV-2 [30,31]. Furthermore, the spike-receptor binding domain (RBD) sequences of SARS-CoV-2 and SARS-CoV were discovered to be 76 percent similar, and the two viruses’ major proteases were determined to be closely related (96 percent identity) [32,33]. The proteins that are involved in the infection of SARS-CoV-2 are summarized in Table 1.

## 3. Cell Death in Coronavirus Infections

Infection with SARS-CoV-2 causes hyperactivation of the immune system, resulting in a cytokine storm. Which is the main cause of lung injury, multiple organ failure, and critical prognosis [44]. Cell death mechanisms are crucial for maintaining an optimal environment for proper cell function. However, during viral infection, dysregulation of these systems can occur and contribute to disease pathogenetic mechanisms [45]. The E protein of SARS-CoV-2 promotes the nuclear factor kappa B (NF-κB) signaling pathway, which results in the generation and release of inflammatory cytokines [46]. For instance, tumor necrosis factor (TNF) and interferon (IFN) are such cytokines released from immune cells.

### 3.1. Apoptosis

**Figure 1:**
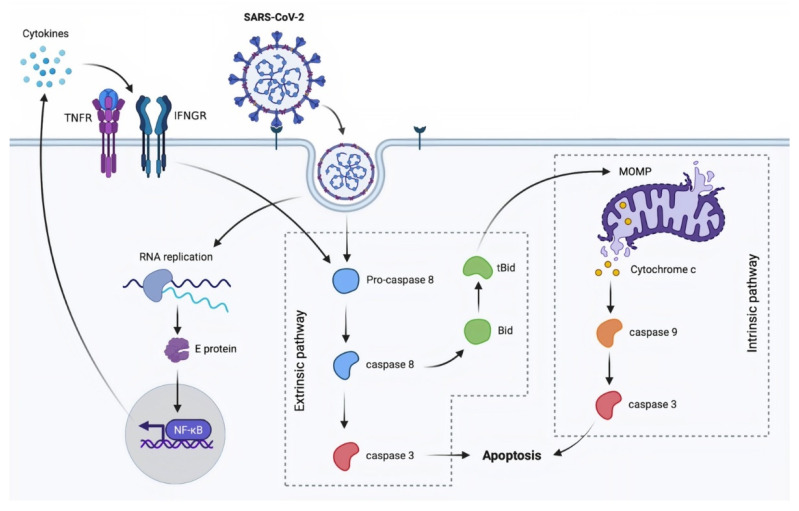
A proposed model of SARS-CoV-2-induced apoptosis via both death receptor and mitochondrial pathways. Bid: BH3 interacting-domain death agonist; caspases: cysteine-aspartic proteases; E protein: envelope protein; IFNGR: interferon-gamma receptor; MOMP: mitochondrial outer membrane permeabilization; NF-κB: nuclear factor kappa-light-chain-enhancer of activated B cells; RNA: ribonucleic acid; SARS-CoV-2: severe acute respiratory syndrome coronavirus 2; tBid: truncated Bid; TNFR: tumor necrosis factor receptors.

SARS-CoV-2 infection activates both intrinsic and extrinsic apoptosis pathway [47,48] (Figure 1). SARS-CoV-2 studies have paralleled SARS-CoV investigations in this sector. Open reading frame 3a (ORF3a), a conserved coronavirus accessory protein, was studied [48,49]. ORF3a is a viroporin, a transmembrane protein, that acts as an ion channel and has been linked to virus release [50,51,52]. In fact, depletion of this protein inhibits viral replication in animal studies [53]. Experiments on several cell lines demonstrated that ORF3a can activate the extrinsic apoptotic pathway via caspase-8 and cross-talk to the intrinsic pathway by cleaving Bid to tBid, resulting in the release of mitochondrial cytochrome c and caspase-9 activation. ORF3a is likewise found in SARS-CoV-2 and shares 73% homology with its SARS-CoV homologue. Mutations that result in a cytosolic form of the protein diminish the ability of SARS-CoV-2 to induce apoptosis, whereas the membrane-associated characteristic is involved but not required in activating this pathway for SARS-CoV. It is indicated that SARS-CoV-2 has weaker pro-apoptotic activity than SARS-CoV and that this distinction may explain some of the differences in pathogenesis between these viruses. Indeed, SARS-CoV-2 is considered to be less virulent than SARS-CoV and to result in the development of asymptomatic patients, which aids in the virus’s widespread transmission [48].

An essential aspect of SARS-CoV-2 infection is a large decrease in CD4+ and CD8+ T-cell subsets that has no effect on the CD4+/CD8+ ratio but has an effect on absolute numbers. Lymphopenia is a risk factor for viral infections and has been linked to disease severity in COVID-19 infection. Although the causes driving lymphopenia remain unexplained, several hypotheses have been proposed [53]. Increased VDAC has been linked to mitochondrial dysfunction and apoptotic gene programs. Dysmorphic mitochondria and cytochrome c release into the cytoplasm were shown by high-resolution fluorescence and electron microscopy imaging of the cells, indicating apoptosis activation. In aged people, the percentage of these cells is significantly higher, and this is linked to lymphopenia. Importantly, T cell death may be decreased in vitro by reducing caspase activity or targeting voltage-dependent anion channel (VDAC) oligomerization [54]. According to another rationale, a cytokine storm can cause a pro-inflammatory state, which causes lymphocyte apoptosis [55]. There is further evidence that CD95/Fas/APO-1 has a role in the activation of apoptosis in SARS-CoV-2 patients’ lymphoid cells. CD95 is a death receptor that belongs to the TNF receptor family and is involved in apoptosis mediated by the extrinsic route [56]. CD95 expression increases in infected participants’ circulating CD4+ and CD8+ lymphocytes compared with healthy controls, and there is a direct link between higher CD95 expression and lower CD4+ absolute count. A similar pattern is discovered in CD8+ T cells [57]. According to a recent study, after RNA-sequencing of PBMCs from COVID-19 patients, several genes are changed, including those associated to apoptosis and P53 signaling; such genes are substantially expressed in COVID-19 patients compared with healthy donors [58].

Single-cell RNA sequencing (scRNA-seq) of CD3+ T cells from COVID-19 patients with acute illness demonstrated an increase in cell death pathway genes. These findings also showed that CD3+ T cells have mitochondrial malfunction, as evidenced by the downregulation of many proteins involved in mitochondrial organization and function, including VDAC and caspases [54]. From electron micrographs, the mitochondria of COVID-19 patients’ lymphocytes are dysmorphic and contain incomplete cristae. Cyt-c is discovered in the cytoplasm of COVID-19 patients’ lymphocytes, suggesting the presence of damaged or dysfunctional mitochondria. This intracellular situation also indicates the activation of the intrinsic apoptotic pathway [53].

### 3.2. Necroptosis

**Figure 2:**
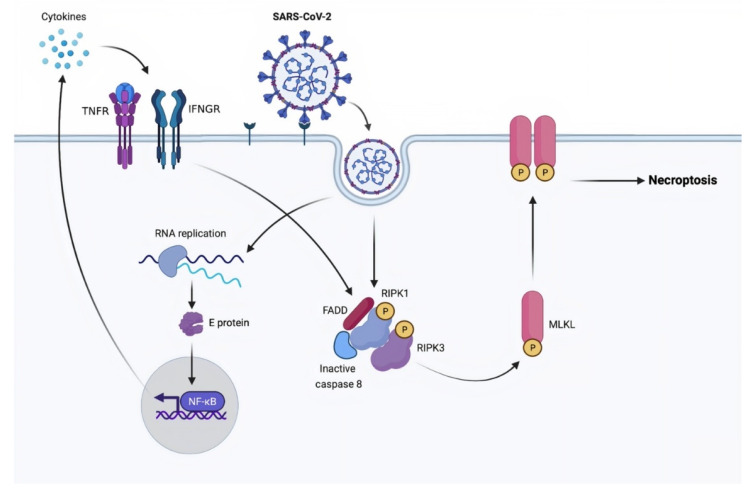
A proposed model of SARS-CoV-2-induced necroptosis. Caspases: cysteine-aspartic proteases; E protein: envelope protein; FADD: Fas-associated via death domain; IFNGR: interferon-gamma receptor; MLKL: mixed lineage kinase domain-like pseudokinase; NF-κB: nuclear factor kappa-light-chain-enhancer of activated B cells; RIPK1: receptor-interacting serine/threonine-protein kinase 1; RIPK3: receptor-interacting serine/threonine-protein kinase 3; RNA: ribonucleic acid; SARS-CoV-2: severe acute respiratory syndrome coronavirus 2; TNFR: tumor necrosis factor receptors.

Calu-3, a human airway epithelial lung cancer cell line, was used to test whether SARS-CoV-2 can cause necroptosis. Mixed lineage kinase domain-like pseudokinase (MLKL) functions as a necroptosis effector that is phosphorylated by receptor-interacting serine/threonine-protein kinase 3 (RIPK3). The phosphorylation of MLKL is examined using Western blotting and immunofluorescence when Calu-3 is infected with SARS-CoV-2. The infected cells have higher levels of pMLKL, and the staining pattern suggested that this protein is present on the plasma membrane. The infected cells are activated with UV light to confirm that the process is driven by SARS-CoV-2. This procedure inhibits virus proliferation and MLKL phosphorylation, demonstrating viral-dependent necroptotic pathway activation. Furthermore, inhibiting RIPK3 in infected cells decreases pMLKL, showing that SARS-CoV-2 activates necroptosis via RIPK3 [11]. Ripoptosome is one of the key regulators of RIPK3–MLKL-dependent necroptosis signaling pathways [59]. It consists of the core components caspase-8, Fas-associated via death domain (FADD), and RIPK1. In the absence of caspase-8 activation, RIPK1 phosphorylates RIPK3 and then promotes necroptosis via MLKL phosphorylation [60] (Figure 2).

### 3.3. Pyroptosis

**Figure 3:**
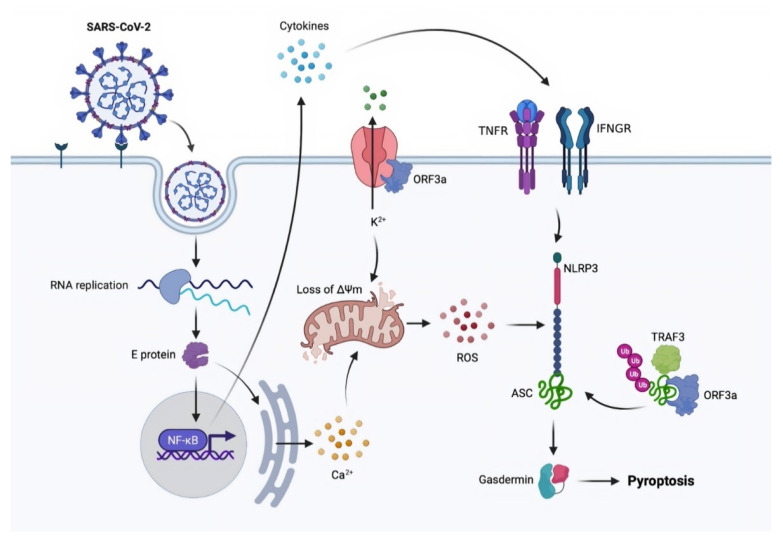
A proposed model of SARS-CoV-2-induced pyroptosis. ASC: apoptosis-associated speck-like protein containing a CARD; Ca^2+^: calcium; E protein: envelope protein; IFNGR: interferon-gamma receptor; K^2+^: potassium; NF-κB: nuclear factor kappa-light-chain-enhancer of activated B cells; NLRP3: NLR family pyrin domain containing 3; ORF3a: open reading frame 3a; RNA: ribonucleic acid; ROS: reactive oxygen species; SARS-CoV-2: severe acute respiratory syndrome coronavirus 2; TNFR: tumor necrosis factor receptors; TRAF3: TNFR-associated factor 3; ΔΨm: mitochondrial membrane potential.

The involvement of the inflammasome in pyroptosis activation is well understood. SARS-CoV has at least three proteins that can activate NLR family pyrin domain-containing 3 (NLRP3), including viral envelope (E) protein, ORF8b, and ORF3a. Because NLRP3 is ionic concentration sensitive, the virus appears to act via its E-protein viroporin, allowing Ca2+ leakage into the cytosol and ORF3a-mediated extracellular K+ efflux. Ionic imbalance causes mitochondrial injury and ROS production, both of which coactivate NLRP3 [61,62]. E protein and ORF3a can activate NLRP3 by (i) activating NF-κB signaling, which activates the transcription of various inflammatory chemokines and cytokines and (ii) enhancing TNFR-associated factor 3 (TRAF3)-mediated ubiquitination of apoptosis-associated speck-like protein containing a CARD (ASC) [63,64,65,66]. The mechanism of SARS-CoV-2 to activate NLRP3 is currently unknown. However, because ORF3a and E have 73 percent and 94.7 percent homology to SARS-CoV, respectively, it is possible that SARS-CoV-2 uses the same, or at least similar, processes [67] (Figure 3).

The major receptor for SARS-CoV-2, ACE2, has been discovered on the surface of many cell types. Its function is to convert angiotensin II (Ang II) to angiotensin 1–7 [68]. These proteins are members of the renin–angiotensin–aldosterone system (RAAS), although their effects are diametrically opposed. The former binds to the angiotensin 1 receptor (AT1R), whereas the latter stimulates the Mas Receptor (MasR). The activation of AT1R after SARS-CoV-2 infection has negative consequences such as fibrosis induction and an increase in reactive oxygen species (ROS) production. Its hyperactivation may also result in the activation of the NLRP3 inflammasome and cell death via pyroptosis [69]. After binding to SARS-CoV-2, ACE2 is internalized and, as a result, does not complete the conversion of angiotensin II to angiotensin 1–7, resulting in AT1R stimulation and NLRP3 activation [70]. The NLRP3 Inflammasome has been targeted in severe COVID-19 [71].

### 3.4. Autophagy

**Figure 4:**
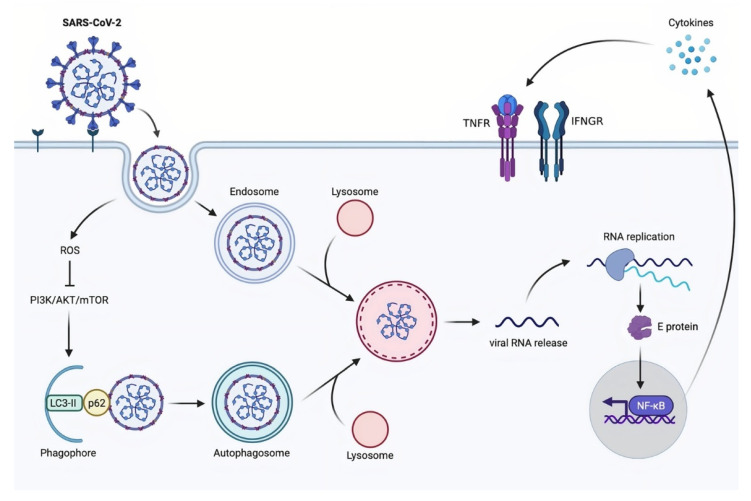
A proposed model of SARS-CoV-2-induced autophagy. AKT: serine/threonine kinase 1; E protein: envelope protein; IFNGR: interferon-gamma receptor; LC3-II: the conjugation of LC3 (microtubule-associated protein light chain 3) to phosphatidylethanolamine; mTOR: mammalian target of rapamycin; NF-κB: nuclear factor kappa-light-chain-enhancer of activated B cells; p62: sequestosome 1 (SQSTM1); PI3K: phosphoinositide 3-kinases; RNA: ribonucleic acid; ROS: reactive oxygen species; SARS-CoV-2: severe acute respiratory syndrome coronavirus 2; TNFR: tumor necrosis factor receptors.

Autophagy is a catabolic mechanism that has a variety of roles in maintaining cellular homeostasis [72]. At numerous levels, this process is implicated in the antiviral response, including the direct removal of intruding viruses (virophagy), the presentation of viral antigens, immune cell fitness, and the prevention of excessive inflammatory reactions. Viruses have evolved methods to interfere with or avoid the autophagic process, and in some cases, even to harness autophagy or components of the autophagic apparatus for their replication, in keeping with its major function in immunity [73]. At this time, it is impossible to establish whether autophagy induction will be beneficial in combating SARS-CoV-2 infection specifically. However, despite some inconsistent results, available data on other CoVs show that autophagy induction could be a viable strategy that should be investigated further. During infection with many CoVs, including SARS-CoVs, the autophagy–lysosomal system appears to play a key role in infected epithelial cells [74], Middle East respiratory syndrome coronavirus (MERS-CoV) multiplication [75]. The SARS-CoV-2 spike pseudovirions (SCV-2-S) generated from the spike-expressing virus packaging technique was used to investigate the regulation mechanism of SARS-CoV-2 in autophagic response. SCV-2-S infection triggers autophagy in human bronchial epithelial and microvascular endothelial cells via the angiotensin-converting enzyme 2 (ACE2) receptor binding. SCV-2-S induces autophagy mediated by upregulating intracellular reactive oxygen species (ROS) levels and inhibiting the PI3K/AKT/mTOR pathway. Infected host cells with SCV-2-S eventually experience inflammatory reactions as a result of SCV-2-S-induced autophagy [76] (Figure 4).

### 3.5. PANoptosis

When TNF and interferon gamma (IFN-γ) attach to their receptors, they can activate inflammatory cell death, PANoptosis: (1) pyroptosis via cleavage of inactive full-length gasdermin into its active form making it capable of forming membrane pores; (2) apoptosis via the extrinsic pathway; and (3) necroptosis by activation of mixed lineage kinase domain-like (MLKL) [77]. However, the roles of cell death in COVID-19 and their mechanisms are still being researched. Increased pro-inflammatory cytokine production and acute lung damage are both associated with patient mortality in COVID-19. In response to SARS-CoV-2 infection, innate immune cells produce a range of inflammatory cytokines, but only the combination of TNF-α and IFN-γ triggers inflammatory cell death, commonly known as PANoptosis. The JAK/STAT1/IRF1 axis is activated by TNF-α and IFN-γ co-treatment, resulting in nitric oxide (NO) production and PANoptosis mediated by caspase-8/FADD. Inhibition of PANoptosis protects mice from pathology and death caused by TNF-α and IFN-γ, which resemble COVID-19’s tissue damage and inflammation. Furthermore, animals given neutralizing antibodies against TNF-α and IFN-γ are protected from death following SARS-CoV-2 infection, sepsis, hemophagocytic lymphohistiocytosis (HLH), and cytokine shock. Inhibiting the cytokine-mediated inflammatory cell death signaling pathway is suggested as a technique to assist COVID-19 patients by reducing tissue damage and inflammation [77].

One possible link between cytokine storm and organ damage is the process of cell death. The most well-studied of the programmed cell death pathways are pyroptosis, apoptosis, and necroptosis. Inflammasome-mediated and gasdermin family members, such as caspase-1 cleavage of gasdermin D (GSDMD), caspase-11/4/5 or caspase-8 cleavage of GSDMD, caspase-3 cleavage of gasdermin E (GSDME), or granzyme cleavage of gasdermin B (GSDMB) are important signaling pathways of pyroptosis [78,79,80,81,82,83,84]. Caspases-3 and -7 carry out apoptosis after upstream initiator caspases (caspase-8/10 or -9) have been activated [85,86]. RIPK3-mediated oligomerization of MLKL produces necroptosis [87,88,89]. Pyroptosis and necroptosis are lytic death processes in which cytokines and other cellular components are released [85,89,90,91].

Cells may experience a lot of crosstalk, which can result in PANoptosis, depending on the stimuli. PANoptosis is a physiologically significant inflammatory-controlled cell death mechanism that is triggered by certain stimuli and regulated by the PANoptosome complex. The PANoptosome is a structure that permits crucial pyroptosis, apoptosis, and necroptosis components to interact with one another [77,90,92,93,94,95,96,97,98].

TNF-α and IFN-γ trigger apoptosis in murine bone marrow derived macrophages (BMDMs), as demonstrated by caspase-3, -7, -8, and -9 cleavage, according to Karki et al. Furthermore, recent investigations have revealed that activation of caspase-3 and -7 can inactivate GSDMD by digesting it to create a P20 fragment [99,100]. Lymphopenia is one of the markers of TNF-α and IFN-γ shock or severe COVID-19 [101]. T cells have been proven to die when exposed to NO, a kind of nitrogen reactive species [102].

Increased pro-inflammatory cytokine production and acute lung damage are both associated with patient mortality in COVID-19. In response to SARS-CoV-2 infection, innate immune cells produce a range of inflammatory cytokines and trigger inflammatory cell death—namely, PANoptosis. The JAK/STAT1/IRF1 axis is activated by TNF-α and IFN-γ co-treatment, resulting in NO production and PANoptosis mediated by caspase-8/FADD. Inhibition of PANoptosis protects the animal model from pathology and death caused by TNF-α and IFN-γ, which resemble COVID-19’s tissue damage and inflammation. Furthermore, the animals given neutralizing antibodies against TNF-α and IFN-γ are protected from death following SARS-CoV-2 infection, sepsis, HLH, and cytokine shock. Inhibiting the cytokine-mediated inflammatory cell death signaling pathway is suggested as a technique to assist COVID-19 patients by reducing tissue damage and inflammation [77].

Taken together, TNF-α and IFN-γ work together to cause the cytokine storm and cell death linked to COVID-19 and sepsis. It was highlighted that (1) only synergism of TNF-α and IFN-γ induces PANoptosis, (2) TNF-α and IFN-γ-mediated PANoptosis prolongs the cytokine storm, (3) TNF-α and IFN-γ shock mimics cytokine storm symptoms, including COVID-19, and (4) TNF-α and IFN-γ neutralization protects such model of mice from SARS-CoV-2, hemophagocytic lymphohistiocytosis (HLH), and sepsis [77].

The discovery downstream of this inflammatory cell death pathway mediated by TNF-α and IFN-γ resulted in the identification of multiple drug targets for COVID-19 and other infectious or inflammatory diseases involving cytokine storm. During COVID-19, researchers have found the processes underlying disease pathology and identified many therapeutic targets in the TNF-α- and IFN-γ-mediated cell death pathway. PANoptosis fills a critical unmet need and serves as a foundation for the development of evidence-based therapeutic strategies to mitigate this ongoing public health crisis.

## 4. The Possible Mechanisms of Cell Death Regulation in SARS-CoV-2 Infection

In genomic sequences, the catalytic sites of the four SARS-CoV2 enzymes are strikingly similar to those of SARS-CoV and MERS. Furthermore, among the three coronaviruses, the architectures of the main drug-binding pockets are remarkably conserved [103]. As a result, existing anti-SARS-CoV and anti-MERS medications that target these enzymes can be repurposed to treat SARS-CoV-2. According to Chen et al. in 2020, the SARS-CoV-2 genome encodes four structural proteins, sixteen non-structural proteins (nsp), and auxiliary proteins, identical to SARS and MERS. Spike (S), envelope (E), membrane (M), and nucleoprotein (N) are all structural proteins that have been used to build anti-COVID-19 medicines [104].

SARS-CoV is thought to have a two-part pathogenic mechanism: (1) the virus directly injures target cells, and (2) causes immune system malfunction. T cells and macrophages are the most important cells in this step. These circulating immune cells subsequently carry the viruses to other tissues, including secondary lymphoid organs. Because SARS-CoV is similar to HIV, both viruses assault immune cells and produce immunodeficiency [105]. Normally, pattern recognition receptors in the host detect viral pathogen-associated molecular patterns (PAMPs). However, the virus can utilize a variety of tactics to evade the innate immune response. The nuclear factor-κB (NF-κB) pathway stimulates the production of type I IFN. Signal transducer and activator of transcription (STAT) proteins are activated when IFN binds to the IFN receptor, increasing the synthesis of other antiviral proteins and then blocking SARS-CoV replication [106]. MERS-cell CoV receptor DPP4 is widely expressed on epithelial cells in the prostate, alveoli, kidneys, liver, and small intestine, as well as on active leukocytes. Hence, MERS-tissue CoV’s tropism is broader than any other coronavirus [107]. The clinical antiviral drugs that target structural and nonstructural proteins of SARS-CoV-2 are employed to develop the following drugs. Lopivir, ritonavir, darunavir, cobicistat, and ASC09F are the most likely inhibitors of 3CLpro (phase III, in combination with oseltamivir, for SARS-CoV-2) [108]. Among the possible inhibitors of RdRp are favipiravir, ribavirin (randomized study for SARS-CoV-2), and remdesivir [109]. Previously, chemicals such as bananins that may interfere with ATPase and helicase activity have been reported (before COVID-19). Another possibility is the creation of antibodies to disrupt the ACE2 receptor, which has a considerable affinity for the RBD of SARS-CoV-2. The second way is blocking the spike protein directly with a significant dose of soluble ACE2. The third way is developing a medicine that directly blocks the spike membrane fusion process, such as enfuvirtide. The fourth strategy is to find an agent that inhibits the action of disintegrin and metalloproteinase 17 (ADAM17), a proteinase that plays a role in the formation of fibrosis or scar tissue [110].

Because SARS-CoV-2 is the third virus of its kind to cause an outbreak, and because it shares similarities with the previous two viruses, SARS-CoV-1 and MERS-CoV, it is critical to determine whether SARS-CoV-2-induced lymphopenia or lymphocyte killing mechanism is the same. COVID-19-associated lymphopenia is a significant pathological finding and severity criterion that can be used as a biomarker and a target for intervention to reduce the likelihood of severe disease. The transition of lymphocyte subsets and variations in peripheral lymphocyte numbers could point to plausible pathways in the pathophysiology of SARS-CoV-2 infection. Only a few studies have looked into severe COVID-19 cases, which have a specific profile of reduced memory T cells. Flow cytometry was used to assess the levels of peripheral lymphocyte subsets in 60 individuals with COVID-19 over the course of their illness. There was a decrease in total lymphocytes, CD4+ T cells, CD8+ T cells, B cells, and natural killer (NK) cells [55]. In a previous study evaluating laboratory indices, twelve patients with COVID-19 were found to have a lower percentage of lymphocytes and a lower CD8 cell count [111]. T cells were found to be more affected by SARS-CoV-2 in 286 patients with COVID-19, with T cell counts about half the lower reference level and tending to be more reduced in severe cases [112]. In a study of severely ill SARS-CoV-2 pneumonia patients, 85 percent of the patients had lymphopenia [113].

The processes underlying the substantial lymphocyte depletion seen in severe instances are established. In COVID-19, if the kind of lymphocyte death and its mechanisms are better understood, it can be a treatment option for severe cases. SARS and MERS have been linked to lymphopenia, which has been linked to apoptosis in the liver, lung, and T lymphocytes [114]. The role of apoptosis in lymphopenia patients with SARS was studied by looking at plasma soluble Fas-ligand levels and cleaved caspase-3 activation in fifteen individuals. In the acute phase of SARS, patients with greater plasma Fas-ligand levels were shown to have more intracellular cleaved caspase-3–positive CD4 and CD8 cells [115].

Apoptosis of lymphocytes is thought to cause lymphopenia in SARS-CoV-2-infected critically sick individuals. It is worth noting that evidence is mounting for direct SARS-CoV-2 infection of T cells, which could result in a cytopathic effect on infected T cells. COVID-19 was found to possess a decrease in the number of circulating Treg cells (CD3+CD4+CD25+CD127low+) [112]. A large drop in lymphocyte numbers, as well as their fast depletion, can play a role in the etiology of COVID-19 and contribute to its development to severe COVID-19. As a result, medications that target lymphocyte growth or apoptosis, such as interleukin IL-2, IL-7, or programmed cell death protein 1 (PD1/PD-L1) inhibitors, may be able to assist in avoiding lymphopenia or in restoring lymphocyte levels in very ill patients [116].

MERS-CoV causes apoptosis in human kidney cells, lung cells, and primary T lymphocytes, most likely through inducing Smad7 and FGF2. SARS-CoV proteins including ORF3a, ORF3b, ORF7a, ORF8a, ORF9b, and E proteins have been shown to be pro-apoptotic proteins. ORF7a promotes the intrinsic apoptotic pathway in human embryonic kidney cell line by interacting with anti-apoptotic protein Bcl-XL in the endoplasmic reticulum, thus sequestering a crucial suppressor of apoptosis. Because MERS and SARS-CoV-2 have similar structural and non-structural proteins in infecting renal and lung epithelial cells, and all types of cells have the cell surface receptors for viral S protein binding and signaling, it is thought that the apoptotic cell death pathway is responsible for all T lymphocytes, kidney cells, and lung epithelial cells [117].

The structural proteins and signals of MERS-CoV, SARS-CoV-1, and SARS-CoV-2 are all similar. Through intrinsic and extrinsic signaling mechanisms, MERS-CoV can infect T cells and trigger apoptosis, bypassing the immune system and allowing for fast spread [118]. SARS-CoV2 may cause T cell cytotoxicity by causing apoptotic-regulated cell death. One method of bypassing the host cell’s immune system has been discovered by MERS-CoV. When host sensors engage signaling pathways, type I IFN genes are transcribed, and the type I IFN response, a key component of antiviral innate immunity, begins. IFN activates the transcription of numerous IFN-stimulated genes (ISGs) and produces 2′,5′-oligoadenylate, which promotes RNase L (2-5A) [119,120,121]. Activated RNase L can cleave both viral and host ssRNA, resulting in translation standstill and death, as well as limiting virus reproduction and transmission [120].

Apoptosis is a well-characterized cell death mechanism caused by viral infection including HIV, MERS, SARS-Co, and SARS-CoV-2, according to various studies [11,48,115,118,122,123,124,125,126]. As a result, because apoptosis includes a vast number of molecules and processes, multiple means for influencing these mechanisms could be applied as follows. Caspase-8 activation initiates an apoptotic cell death via cascades leading to the extrinsic apoptosis pathway. In the extrinsic pathway, FLIP exhibits inhibitory effects on apoptosis.

Since the development of understanding and knowledge of FLIP has a long history of insight and concept concerning the death receptor pathway, it has the following scientific evidence-based data, which is support by much research. The death domain (DD) plays a pivotal role because it is found in the cytosolic part of death receptors, viz., Fas, TNF-R, TRAIL-R1, and TRAIL-R2; adaptor proteins, including FADD (Fas-associated protein with death domain), TRADD (TNF-receptor-associated protein with death domain); and RIP1. Moreover, death effector domain (DED) is in the adaptor proteins, viz., FADD, TRADD, (pro)caspase-8/10, and FADD-like IL-1-converting enzyme (FLICE) [127]. Hence, it was found that in the death-inducing signaling complex (DISC), it comprises death receptor and adaptor protein(s) binding via DD, together with (pro)caspase-8/10, leading to autoactivate the procaspase-8/10 themselves to be active caspase-8/10 that further induces caspases-3, 6, and 7, then finally apoptosis. However, in cells that have cFLIP with DED domain, cFLIP binds to DED of pro-caspase-8/10 and inhibits the induction of the extrinsic apoptosis pathway [127,128]. The roles of apoptosis have been found in physiology, such as in development and pathophysiology, e.g., neurodegenerative diseases [129], viral infection [130], and even cancer [131]. Restraining of SARS-CoV-2 viral replication and virus-induced tissue damage through modulation of the DD network may pave the way towards new therapeutic approaches by inhibiting caspase-8 activity, at death-inducing signaling complex (DISC) formation. Therefore, it was assumed that FLIP containing DED may play a pivotal role in apoptosis induction in COVID-19 patient cells/tissues [127].

Ivanisenko et al. (2020) investigated the pathogen penetration and regulating immune response by creating a mouse model that mimicked a regulated cytokine storm. As indicated by important clinical phenotypes, it was uncovered that SARS-CoV-2 induces pandemic, dysregulated anti-pathogen immune responses, known as cytokine release syndrome (CRS), which can produce life-threatening inflammatory illnesses. FLIP, a protein that regulates caspase-8 death pathways, was found to be highly expressed in the myeloid cells of COVID-19 lungs. FLIP regulates CRS via inducing an inflammatory response that is dependent on STAT3. Consistent expression of a viral FLIP homolog in myeloid cells generates a STAT3-linked, progressive, and fatal inflammatory disease in mice that imitates human CRS and includes elevated cytokine output, lymphopenia, lung injury, and multiple organ dysfunctions. Because STAT3-targeting strategies reduce inflammation, immunological issues, and organ failures in these mice, it is feasible that blocking this pathway can reduce CRS’s lethal inflammatory condition [127].

FLICE (FADD-like IL-1-converting enzyme)-like inhibitory proteins (referred to as c-FLIP and v-FLIP) functions as an anti-apoptotic cellular and viral factor capable of reprogramming monocytes [132]. FLIP isoforms are known to inhibit caspase-8 and/or activate NF-κB to regulate cell survival and proliferation [133,134,135,136]. FLIPs, on the other hand, regulate different biological processes depending on their protein structure (for example, the presence of several death effector domains in a single protein) [137], cellular location (for example, nucleocytoplasmic trafficking) [138], and nuclear localization of c-FLIPL and its regulation of AP-1 activity. Upregulation of FLIP proteins in monocytes leads to the development of an unusual phenotype characterized by the expression of immunosuppressive (e.g., programmed death-ligand 1 (PD-L1), interleukin (IL)-10) and pro-inflammatory (e.g., IL-1, IL-6, tumor necrosis factor (TNF)-α) features, which is partially dependent on the nuclear translocation of the complex FLIP/nuclear factor complex [127].

FLIP- and pSTAT3-expressing myeloid cells have also been linked to COVID-19-associated CRS, since both human ACE2-expressing transgenic mice and SARS-CoV-2 patients possess a high level of FLIP. Furthermore, monocytes isolated from COVID-19 patients exhibit high levels of myeloid c-FLIP and pSTAT3, which is linked to their immunosuppressive properties. Immunological dysfunctions and the bronchoalveolar immune landscape of patients with severe COVID-19 were discovered to be reflected in vFLIP transgenic mice. This one-of-a-kind model was used to investigate STAT3 inhibition approaches for treating uncontrolled inflammation and acute sickness symptoms in systemic and myeloid-targeted mice. Finally, it was revealed that a novel pathway of FLIP’s role is its critical function and expression in myeloid cells as a direct means to stimulate a deadly inflammatory state by fueling an abnormal STAT3-dependent signaling pathway. Furthermore, the therapeutic efficacy of the STAT3 on-target strategy in reducing uncontrolled inflammation and acute disease becomes the laying of a groundwork for the development of more precise and evidence-based therapies to treat CRS disorders and severe clinical aspects of the ongoing COVID-19 pandemic crisis. The importance of FLIP and the proteins involved in the extrinsic route in limiting the DD-mediated signaling network to potentially suppress viral replication and reduce tissue damage was highlighted in this study [127].

As mentioned, the participation of cellular FLICE-like inhibitory protein (cFLIP) in the death-inducing signaling complex (DISC) is required for its activation. At the DISC, the long isoform of cFLIP (cFLIPL) can operate in both a pro- and anti-apoptotic manner [139,140]. Because it operates as an anti-apoptotic factor by inhibiting procaspase-8 activation, viruses upregulate cFLIP expression as a strategy to prevent extrinsic apoptosis in order to continue their replication [140]. High amounts of cFLIP were found in the pulmonary myeloid cells of COVID-19 patients as well as during the start of SARS-CoV-2 infection [141]. As a result, the use of small molecules that act against cFLIP to activate caspase 8 and induce extrinsic apoptosis has been proposed as an important strategy for combating virus replication [128]. 4-(4-Chloro-2-methylphenoxy)-N-hydroxybutanamide (CMH) and droxinostat, the two small molecule inhibitors of cFLIP, have been found to reduce c-FLIPL and c-FLIPS mRNA and protein levels, which can inhibit cell viability and promote apoptosis [131]. Inhibiting apoptosis in T cells by using small molecules such as pancaspase inhibitor (z-VAD-fmk) or TRADD inhibitors (ICCB-19 and Apt-1) [142] seemed to be an appealing strategy for preventing lymphopenia in COVID-19 patients [55].

Furthermore, either VDAC1 oligomerization inhibitors (VBIT-4, VBIT-3, and AKOS-022) [143] or a pan-caspase inhibitor may rescue the survival of T cells that showed characteristics of apoptosis caused by mitochondrial degeneration as well [5]. These findings are consistent with the fact that VDAC1 oligomerization and interaction with Bcl-2 family proteins are thought to be responsible for the formation of pores in the outer membrane of mitochondria, allowing for cytochrome c release and the activation of the caspase cascade, which induces cellular apoptosis [144,145].

The NF-κB pathway is another important anti-apoptotic route mediated by SARS-CoV-2. When NF-κB is activated, apoptosis inhibitors such as cFLIP, B-cell lymphoma 2 (Bcl-2), and X-linked inhibitor of apoptosis protein (XIAP) are upregulated. This strategy is critical for promoting viral infection, survival, and inflammation [146,147]. Therefore, targeted inhibition of the NF-κB signaling with small molecule drugs such as proteasome inhibitors suggests yet another potential strategy for combating SARS-CoV-2 [148]. Celastrol and curcumin are natural compounds that disrupt the ubiquitin–proteasome system (UPS), which inhibits the NF-κB pathway [149]. In addition, there are several small molecule inhibitors of the NF-κB pathway from cell surface receptor stimulation to nuclear signaling that were approved by the US FDA. Acalabrutinib, a Bruton’s tyrosine kinase (BTK) inhibitor, and selinexor, a selective inhibitor of nuclear export (SINE) acting via NF-κB inactivation, are currently being evaluated in COVID-19 clinical trials [150,151,152,153].

The use of necrostatin-1 (Nec-1) and Nec-1 analogues may relieve cytokine storm, systemic inflammation, and COVID-19 infection by inhibiting RIPK1, the molecule that interacts with RIPK3 forming rippoptosome and promotes MLKL phosphorylation [154]. Because RNA viruses can activate the inflammasome via RIPK1/RIPK3 [155], Nec-1 may aid in the regulation of this process by decreasing inflammation and cytokine release caused by COVID-19. To prevent necroptosis, researchers used the RIPK1-specific inhibitor necrostatin-1 (Nec-1), RIPK3 inhibitor GSK’872, MLKL inhibitor necrosulfonamide (NSA), and small interfering RNA (siRNA) [156]. It is unclear whether inhibiting necroptosis improves the host’s antiviral response or worsens tissue inflammation and damage. In fact, necroptosis can both inhibit viral replication by causing cell death and can enhance viral dissemination by causing host cell rupture.

SARS-CoV-2 activates the inflammasome and causes pyroptosis in human monocytes. As a result, the use of MCC950, a potent and specific inhibitor of the NLRP3 inflammasome, may alleviate cytokine storm and systemic inflammation caused by SARS-CoV-2-induced immunogenic cell death [157,158].

In different disease models, such as cancer [159], neurological illnesses, cardiovascular disease, and inflammatory diseases [160], several plant extracts such as *Tiliacora racemosa* leaf methanolic extract and natural constituents such as fisetin and quercetin can inhibit the controlled apoptotic cell death pathways by targeting multiple death-inducing molecules. As a result, we hypothesize that the plant-derived phyto-chemicals may have a similar effect on viral infection-mediated apoptotic diseases (including SARS-CoV2).

The FDA-approved anti-malarial medications chloroquine and hydroxychloroquine have been proposed for the treatment of COVID-19 via autophagy inhibition [161,162,163]; however, this is extensively debated [164,165]. Although chloroquine is a lysosomotropic drug that inhibits autophagic degradation, potentially by inhibiting autophagosome fusion with lysosomes [166], the putative autophagic effects may not be responsible for the antiviral action. Indeed, endosomal acidification following endocytosis is required for SARS-CoV-2 entry [9], and chloroquine suppresses this acidification [167]. Furthermore, chloroquine inhibits the terminal glycosylation of the metallopeptidase ACE2, which serves as a functional receptor for SARS-CoV and SARS-CoV-2 cell entry [7,9]. Non-glycosylated ACE2 appears to interact with the SARS-CoV spike protein less efficiently, leading to a decrease in viral entry [168].

## 5. The Therapeutic Targets against Coronavirus

There are various specific medications for tackling this virus, and the process of developing novel and more effective drugs is ongoing. In hospitalized adult patients with mild or severe non-ICU COVID-19, canakinumab can be a valid therapeutic option. Canakinumab therapy causes rapid and long-lasting improvement in oxygenation levels in the absence of any severe adverse events [169]; tocilizumab for treatment of SARS-CoV-2 pneumonia has also been reported [170,171]. In patients infected by the SARS-CoV-2 gamma variant, bamlanivimab/etesevimab should be used with caution because of the high risk of disease progression [172]. Currently, there are numerous drugs based on various rationales and strategies used for COVID-19 therapy in clinical trials [173].

The majority of promising candidates that emerged as prospective leads were developed in the early stages of clinical trials based on the properties of structural and non-structural proteins. However, natural products are widely used arbitrarily as anti-viral drugs and immune boosters in the absence of definite therapies. For centuries, it has been recognized that most natural compounds have significant anti-viral activity, and SARS-CoV-2 is not an exception [174,175].

Natural products are produced by an organism’s metabolic processes, but according to the contemporary perspective of pharmacology, people use them for their health benefits. Various medicinal herbs with anti-inflammatory, antifungal, and anticancer effects have been thoroughly investigated in order to identify their antiviral capabilities—for example, anthocyanins [176] and several other compounds have been found to limit the infection, progression, and amplification of coronaviruses (Table 2). Natural compounds have antiviral properties against coronaviruses by targeting on different molecules in viral entry, amplification, replication, protein synthesis, etc.

For example, at the viral surface, the spike (S) glycoprotein is the most prominent antigen. It is responsible for host cell attachment and mediates host cell and viral membrane fusion during infection [7,177], altering the viral attachment and penetration process [178]. Various natural compounds have the ability to act on viral S glycoprotein. For example, emodin, a main compound of *Rhei Radix* et Rhizoma, has been shown to have antiviral properties against SARS-CoV by targeting S protein and preventing S protein binding to ACE2 in a dose-dependent manner [179,180]. Extracts of *Eucalyptus globulus* and *Lonicera Japonica* Flos have capacity to disrupt the envelope glycoprotein processing. Ginsenoside-Rb1 has the same ability as well [181,182]. Saikosaponin B2 has substantial anti-coronavirus activity by disrupting viral glycoproteins and thereby altering the viral attachment and penetration process [178].

TMPRSS2 is essential for infection of SARS-CoV-2. Activation of TMPRSS2 enhances viral attachment to host cells as well as induces viral and plasma membrane fusion. Hence, TMPRSS2 is also a therapeutic target. Platycodin D from *Platycodon grandiflorum* effectively blocks SARS-CoV-2 infection via TMPRSS2 [183]. In computer modeling, NPC306344, a low-molecular-weight natural product and other six compounds including neohesperidin, myricitrin, quercitrin, naringin, icariin, and ambroxol interact significantly with the active site residues of TMPRSS2 [184,185].

3CLpro and PLpro are essential proteases for the replication and packaging of new viruses by processing the polypeptide translation product from the genomic RNA into the structural and nonstructural protein components [186,187]. Because of the importance of 3CLpro and PLpro in the life cycle of a virus, they are promising targets for the development of anti-SARS-CoV drugs. Research has found that various natural compounds display inhibitory effects against SARS-CoV by inhibiting the activity of 3CLpro, such as chalcones, flavanones and coumarins from *Angelicae Sinensis Radix*, hesperetin and sinigrin from *Isatidis Radix* [188] and celastrol, pristimererin, tingenone, and iguesterin isolated from *Triterygium regelii* [189] and quercetin-3-β-galactoside from *Cercis canadensis* L. [190]. Additionally, in silico investigations have identified andrographolide from *Andrographis paniculata* as a possible inhibitor of the major protease of SARS-COV-2 (Mpro) [191]. Tanshinone I, which was isolated from *Salvia miltiorrhiza* and hirsutenone, which was obtained from *Alnus japonica*, both have dose-dependent inhibitory effects against SARS-CoV via targeting PLpro [192,193].

RdRp, also known as RNA replicase, is a key polymerase that catalyzes the replication of RNA from RNA template. RdRps play a pivotal role in genome replication, mRNA synthesis, and RNA recombination in a variety of RNA viruses. They are necessary for viral survival [194]. RdRp has proven to be a good antiviral target for coronaviruses, and several intriguing compounds have antiviral effects by targeting RdRp—for example, theaflavin [33]. Moreover, sesamin, sesamolin, pinoresinol, hydroxymatairesinol, spicatolignan, ferulic acid, and vanillic acid were tested in silico against three major proteins of SARS-CoV-2, including, 3CLpro, PLpro and RdRp [195]. Moreover, a virus replication enzyme, helicase or Nsp13, is involved in the unwinding of viral RNA. Myricetin and scutellarein isolated from *Isatis indigotica* Fort. and *Torreya nucifera* L., respectively, inhibit SARS-CoV helicase via ATPase activity [196,197].

**Table 2:** Natural products with anti-coronaviral activity targeted on viral machinery.

Group	Compounds	Isolated From	Coronavirus Type	Target	IC_50_	References
**Alkaloids**	Lycorine	*Lycoris radiata*	SARS-CoV	RdRp	1.021 μM	[198]
			SARS-CoV-2		0.878 μM	
	Indigo	*Isatis indigotica*	SARS-CoV	3CLpro	752 μM	[181,188,199]
	Sinigrin				217 μM	
	Reserpine	*Rauvolfia serpentina*	SARS-CoV	3CLpro, PLpro	3.4 μM	[182]
			SARS-CoV-2		5.7 μM	[200]
	Cepharanthine	*Stephania japonica*	SARS-CoV-2	S protein	0.98 μM	[201,202]
**Flavonoids**	Hesperetin	*Isatis indigotica*	SARS-CoV	3CLpro	60 μM	[188,203]
			SARS-CoV-2	S protein	16.88 mM	[204]
	Amentoflavone	*Selaginella sinensis, Torreya nucifera*	SARS-CoV	3CLpro	8.3 μM	[205,206]
	Myricetin	*Myricaceae, Anacardiaceae, Polygonaceae, Pinaceae, Primulaceae*	SARS-CoV	Nsp13, 3CLpro	2.71 μM	[197,207]
	Scutellarein				0.86 μM	
	Scutellarein	*Scutellaria*	SARS-CoV-2	3CLpro	5.8 μM	[208]
	Baicalin	*Scutellaria baicalensis*	SARS-CoV-2	3CLpro	83.4 µM	[208]
	Baicalein				0.39 μM	
	Dihydromyricetin	*Eriocaulon buergerianum*	SARS-CoV-2	3CLpro	1.24 μM	[208]
	Quercetagetin	*Polygoni avicularis*	SARS-CoV-2	3CLpro	2.86 μM	
	Myricetin	*Ampelopsis japonica*	SARS-CoV-2	3CLpro	1.20 μM	
	Scutellarein	*Scutellaria, Erigeronti*	SARS-CoV-2	3CLpro	5.8 μM	
	Tannic acid	*Camellia sinensis*	SARS-CoV	-	3 μM	[209]
			SARS-CoV-2	Mpro, TMPRSS2	2.31-13.4 μM	[210]
	Theaflavin	*Camellia sinensis*	SARS-CoV-2	3CLpro	8.44 μg/ml	[211]
	Epigallocatechin gallate (EGCG)				7.58 μg/ml	
	Papyriflavonol	*Broussonetia papyrifera*	SARS-CoV	PLpro	3.7 μM	[205,212,213]
	C-5-alkyl group (prenyl)-substituted flavan			3CLpro	52.7 μM	
	Xanthoangelol E	*Angelica keiskei*	SARS-CoV	PLpro, 3CLpro	11.4 μM	[214]
**Terpenoids**	Glycyrrhizin	*Glycyrrhiza glabra*	SARS-CoV-2	Mpro	0.44 mg/mL	[215]
	Betulinic acid	*Betula pendula, Pterocarpus santalinus*	SARS-CoV	3CLpro	10 μM	[216]
	Savinin				25 μM	
	Betulinic acid	*Olea europaea*	SARS-CoV-2	Mpro	14.55 μM	[217]
	Andrographolide	*Andrographis paniculata*	SARS-CoV-2	Mpro	0.034 μM	[191,218]
**Diarylheptanoids**	Hirsutenone	*Alnus japonica*	SARS-CoV	PLpro	4.1 μM	[193]
	Hirsutanonol				7.8 μM	
	Oregonin				20.1 μM	
	Rubranol				12.3 μM	
	Rubranoside B	*Alnus japonica*	SARS-CoV	PLpro	8.0 μM	[193]
	Rubranoside A				9.1 μM	
	Panduratin A	*Boesenbergia rotunda*	SARS-CoV-2	-	0.81 μΜ	[219]
**Anthraquinone** **s**	Emodin	*Rheum, Polygonaceae, Isatis indigotica*	SARS-CoV	S protein	200 μM	[179,220,221]
			SARS-CoV-2	Mpro, S protein, RdRp	-	[222]
	Aloe-emodin		SARS-CoV	3CLpro	132 μM	[188]
			SARS-CoV-2	Mpro, S protein, RdRp	-	[222]

- The data are not clear.

Viruses upregulate anti-apoptotic protein expression as a strategy for preventing host cell apoptosis during the early stages of infection, allowing them to continue replication. [143]. As a result, the use of small molecules capable of inducing apoptosis has been proposed as an important strategy for combating virus replication [123]. According to the existing results, aerodigestive and lung cancer cell lines have a wide range of ACE2 and TMRPSS2 expression and would be ideal models for researching SARS-CoV-2 infection [223]. Hence, human lung cancer cell lines (e.g., Calu-3 and A549) are frequently utilized in the research of respiratory infections including SARS-CoV-2 [223,224]. There are many phyto-chemicals that can induce apoptosis in human lung cancer cell lines (Table 3). As a result, we hypothesize that the death-inducing effect of phyto-chemicals may hinder viral replication during the early stages of infection.

For other kinds of cell deaths (autophagy, necroptosis, and pyroptosis), the investigations on the effects of natural products on the viral inhibition is still elusive and remains in need of further investigation. However, in the same analogy, we expect the herbal constituents or extracts or products will contain the inhibitory effects on viruses, especially CoV or SAR-CoV, positively as being affected in apoptotic cell death induction. Nonetheless, further scientific evidence is still needed to provide data and information.

Because there are no established treatment options for the ongoing COVID-19 epidemic, understanding the mechanism of multiorgan failure and lung injury in these patients is critical. Given the association between cytokine storm and severe inflammation and organ damage during COVID-19, and the lack of a precise molecular pathway defining cytokine storm, acquiring a mechanistic understanding is critical for creating treatments.

## 6. Conclusions

Globally, SARS-CoV-2 is a virus that causes severe COVID-19 pandemic fatalities. Extensive research is being performed to investigate drugs or natural products for prophylaxis and treatment, while the vaccine development is still developing simultaneously to prevent the infection. The viruses cause host cell death and inflammation due to the cytokine storm. Numerous medicinal herbs and the phyto-chemicals possess anti-inflammatory and antiviral effects on the progression and amplification of numerous viruses. Natural compounds targeting against coronaviruses including SARS-Co-V2 via different molecules involving in the processes of viral entry, amplification, replication, and protein synthesis were provided in detail. Such components included alkaloids, flavonoids, terpenoids, diarylheptanoids, polyphenolic acids, saponins, and anthraquinones from nature with fewer unfavorable side effects, as expected. The targeted molecules of SARS-CoV-2 for infection are as follows: spike (S), TMPRSS2, papain-like proteinase (PLpro), viral main protease (3C-like protease (3CLpro or Mpro)), non-structural protein 13 (Nsp13) helicase, open reading frame 7a (ORF7a), and RNA-dependent RNA polymerase (RdRp) (Nsp12), which are the proteins needed for making new virions because the host cells are killed by five different types of regulated cell death, viz., apoptosis, necroptosis, pyroptosis, autophagy, and PANoptosis. The natural inhibitors or small molecules modifying the cell death pathways or signalings may be of high efficacy for the treatment and prevention of SARS-CoV2 infection. For example, pancaspase inhibitor (z-VAD-fmk) (for apoptosis intervention); necrostatin-1 (a necroptosis inhibitor), MCC950, a specific small-molecule inhibitor of the NLRP3 inflammasome (as a pyroptosis inhibitor); and chloroquine/hydroxychloroquine (as an autophagy inhibitors) may be beneficial. The NF-κB pathway is another important anti-apoptotic and inflammatory route mediated by the viruses since NF-κB signaling promotes viral proliferation, survival, and inflammation via the enhancing of anti-apoptosis proteins such as Bcl-2 and XIAP expression and cytokine release, e.g., TNF and interleukin-1. As a result, tiny compounds functioning as controlling the NF-κB-mediated proteasome inhibition, such as celastrol and curcumin, can be applied to target the ubiquitin–proteasome system (UPS), then affecting NF-κB signaling to provide another reasonable method for prophylaxis and therapy of COVID-19 patients. The linkages of cell death signaling, the mechanism of viral entry/replication, and the natural products are still scarce, and research in this field is advancing and progressing at the molecular level to understand more of the mechanisms of phyto-chemicals and natural compounds as cell death inducers of virus-infected host cells and to understand the use of such strategic targets for orchestrating and obtaining an effective high potential treatment and prevention for the rescue of COVID-19 patients. Nevertheless, acalabrutinib (a tyrosine kinase inhibitor) and selinexor (acting through deactivation of NF-κB signaling) have been investigated in clinical trials for COVID-19 therapy and chloroquine and hydroxychloroquine have been approved by the US FDA for SARS-CoV-2 patients. Various natural compounds’ in vitro cytotoxicity effects have been recorded, although animal model and human tests are still lacking. We anticipate that natural products that target multiple cell death signaling molecules will be examined and authorized in clinical trials with supporting scientific data in the near future. Rather than using COVID-19 therapy, the best course of action is to prevent SARS-CoV-2 infection.

## Figures and Tables

**Table 1:** The proteins involved in the infection of SARS-CoV-2.

Proteins	Function	References
Spike glycoprotein (S protein)	Mediate receptor recognition and membrane fusion for viral entry.	[34]
Angiotensin-converting enzyme 2 (ACE2)	Functional cellular receptor on host cell membrane.	[35]
Cathepsin L-cysteine peptidase	Facilitate the cleavage of S protein for activating membrane fusion.	[36]
Transmembrane protease serine 2 (TMPRSS2)	Cleave C-terminal of ACE2 and activate S-protein.	[37]
Nonstructural protein 1 (Nsp1)	Interact with 40S ribosome subunit to induce host mRNA degradation and inhibit type I interferon production.	[38]
Open reading frame 7a (ORF7a)	Block the activity of bone marrow stromal antigen 2 (BST-2) by directly binding and disrupting the glycosylation of BST-2. (BST-2 mediates the restriction of virus-like particle release.)	[39]
Replicase polyprotein 1ab	Transcription and replication of viral RNAs.	[40]
Papain-like proteinase (PLpro)	Cleave the N-terminal of replicase polyprotein leading to release of Nsp2 and Nsp3, which are in turn involved in viral replication.	[41]
Viral main protease (3C-like protease (3CLpro) or Mpro)	Essential for viral replication by controlling the activity of replication complex.	[42]
RNA dependent RNA polymerase (RdRp) (Nsp12)	Catalyze the replication of viral RNA from RNA template.	[33]
Non-structural protein 13 (Nsp13) helicase	NTPase, duplex RNA/DNA-unwinding and RNA-capping activities.	[43]

**Table 3:** Phyto-chemicals inhibiting SARS-CoV-2 infection by using lung cancer models via specific death signaling pathways.

Phyto-Chemicals	Plants	Target Cell Lines	IC_50_	Signaling Pathways	References
Graveospene A	*Casearia graveolens*	A549	1.9 M	Triggers apoptosis by inducing cell cycle arrest in phase G0/G1.	[225]
Licochalcone A	*Xinjiang licorice* and *Glycyrrhiza inflata*	A549, H460, SPC-A1, H23, and H1299	<40 μM	Induces apoptosis by downregulating the expression of anti-apoptotic proteins such as c-IAP1, c-IAP2, XIAP, survivin, and c-FLIPL by inhibiting the activity of phosphorylated extracellular signal-regulated kin (ERK).	[226]
Erianin	*Dendrobium chrysotoxum* Lindl	H460 and H1299	>100 nM	Induces apoptosis by promoting G2/M-phase arrest.	[227]
Erianthridin	*Dendrobium formosum*	H460	150.9 μM	Induces apoptosis by suppressing extracellular signal-regulated kinase activity.	[228]
		A549	161.9 μM		
Gracillin	*Reineckia carnea*	A549	2.54 mol/L	Induces apoptosis via the mitochondrial pathway.	[229]
Hispidulin	*Saussurea involucrate*	A549 and NCL-H460	<30 μM	Promotes apoptosis by increasing ROS production and activating ER stress.	[230]
Liriopesides B (LPB)	*Liriope platyphylla*	H460	42.62 µM	Induces apoptosis by inhibiting the progression of the cell cycle from G1 to S phase.	[231]
		H1975	32.25 µM		

## Data Availability

Data sharing is not applicable to this article.
